# Recent Advances in the Development of Protein- and Peptide-Based Subunit Vaccines against Tuberculosis

**DOI:** 10.3390/cells9122673

**Published:** 2020-12-15

**Authors:** Chiara Bellini, Kata Horváti

**Affiliations:** 1 Hevesy György PhD School of Chemistry, Eötvös Loránd University, 1117 Budapest, Hungary; chiara.bellini@ttk.elte.hu; 2 MTA-ELTE Research Group of Peptide Chemistry, Eötvös Loránd University, 1117 Budapest, Hungary

**Keywords:** *Mycobacterium tuberculosis*, subunit vaccine, protein-based vaccine, peptide-based vaccine, antigen, adjuvant, tuberculosis, epitope

## Abstract

The World Health Organization (WHO) herald of the “End TB Strategy” has defined goals and targets for tuberculosis prevention, care, and control to end the global tuberculosis endemic. The emergence of drug resistance and the relative dreadful consequences in treatment outcome has led to increased awareness on immunization against *Mycobacterium tuberculosis* (*Mtb*). However, the proven limited efficacy of Bacillus Calmette-Guérin (BCG), the only licensed vaccine against *Mtb*, has highlighted the need for alternative vaccines. In this review, we seek to give an overview of *Mtb* infection and failure of BCG to control it. Afterward, we focus on the protein- and peptide-based subunit vaccine subtype, examining the advantages and drawbacks of using this design approach. Finally, we explore the features of subunit vaccine candidates currently in pre-clinical and clinical evaluation, including the antigen repertoire, the exploited adjuvanted delivery systems, as well as the spawned immune response.

## 1. Introduction

According to the WHO’s Report of 2020, around 10 million people fell ill with tuberculosis (TB) each year, and 1.5 million died only in 2018, making it the leading cause of death from a single infectious agent [1].

TB represents an urgent health priority, especially in high-burden countries, where 90% of cases are registered. Most of these countries are low income and developing states in which TB has severe economic, social, and public health consequences. The epidemiological scenario in these countries is worsened by health-related risk factors, like the correlation with HIV in TB-areas, likewise by broader determinants, such as undernutrition and poverty [1]. Additionally, deep concern is currently emerging about the COVID-19 outbreak impingement on TB handling due to the limited access to treatment and diagnosis, as well as the partial overlap of disease symptoms [2]. Despite drug treatments continue to improve, reports of multidrug resistance tuberculosis (MDR-TB) and extensively drug-resistance tuberculosis (XDR-TB) are increasing worldwide, reducing the treatment success rate at unacceptably low levels. The latest available data show an 85% success rate for drug-susceptible TB, 56% for MDR-TB, and 39% for XDR-TB [3]. Nevertheless, the estimated treating cost for MDR-TB ranges from eight-fold to 15-fold higher compared to sensitive-TB, while it can rise to 32-fold higher in XDR-TB cases [1,4,5].

## 2. BCG: A Cornerstone in TB Handling

BCG vaccine is an attenuated form of *Mycobacterium bovis*, the primary cause of bovine tuberculosis, which is genetically related to *Mycobacterium tuberculosis* (*Mtb*). Used for the first time in humans in 1921, it still represents the gold standard in TB vaccination, although several aspects of this vaccine remain incompletely characterized, including its overall efficacy, duration of protective immunity, and how age at vaccination affects the protection [6,7].

The role of BCG in preventing severe forms of extra-pulmonary TB in children, like TB meningitis and miliary TB, has been proved [8,9]. Conversely, a limited protective effect was observed against pulmonary TB, especially in adults, which is not only the most prevalent form of the disease but also the principal source of transmission [10]. Epidemiological evidence highlighted that the BCG degree of protection varies geographically [6,7,8,11,12,13,14,15], while several studies suggested a lower and short-lasting BCG protection in people previously sensitized with *Mtb* and non-tuberculous mycobacteria (NTB) [7,11]. Additionally, it has an unfavorable risk profile in immunocompromised conditions, for example, congenital immunodeficiencies and HIV infections, because of BCG bacteremia that follows vaccination [9,13]. Commonly, BCG has been shown to protect infants and young children up to approximately 10 years of age, although some degree of protection might last for up to 20 years following school-aged BCG vaccination and as long as 50–60 years following infant vaccination [8,9,15]. However, it cannot provide lifelong immunity, and there are no studies that currently demonstrate a reliable effect against TB of BCG revaccination in adolescents [8]. Nevertheless, a Phase II clinical trial is ongoing to demonstrate the efficacy of revaccination against sustained *Mtb* infection in previously BCG vaccinated people (NCT04152161).

Given the scale of the global TB epidemic and the slow pace of TB control efforts, it is clear that a novel and more effective TB vaccine is needed to achieve WHO End TB Strategy targets by reducing TB deaths by 95% and new cases of TB by 90% by 2035 [1].

## 3. Tuberculosis Immunopathology

**Figure 1:**
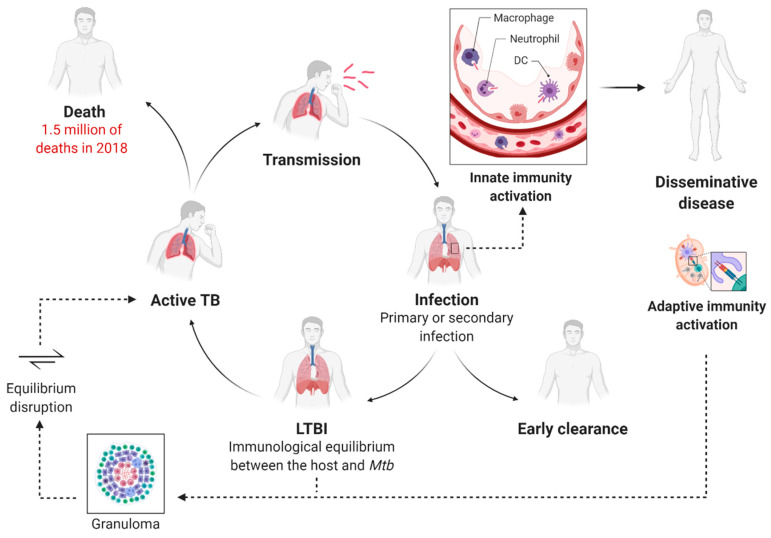
The immunological lifecycle of *Mycobacterium tuberculosis* and the progression of the disease. *Mtb* is commonly aerosol transmitted, and the innate immune response acts as a first barrier in the alveolar space. At the early stage of infection, *Mtb* may be rapidly eradicated. The trafficking of the antigen-presenting cells to the lymph nodes leads to the dissemination of the disease as well as to the activation of the adaptive immune response. T-lymphocytes regulate the formation of the granuloma within *Mtb* is contained, and the individual develops latent TB infection (LTBI). When the immunological equilibrium between the host and the pathogen is disrupted, latent TB evolves to active disease. At this stage, the patient becomes symptomatic and can transmit the infection to healthy individuals.

*Mtb* infection is a complex, multi-stage process that generally arises after the inhalation of *Mtb*-containing aerosolized droplets released from people with active pulmonary TB (Figure 1). The respiratory tract and bronchoalveolar spaces represent a unique immunological compartment where the innate immune system and the respiratory epithelium act as the first barrier to providing early clearance of the pathogen [11,16,17,18,19]. Following initial exposure to *Mtb*, a small percentage of exposed humans can quickly clear the infection. Besides, a growing number of studies evidence that a subset of people does not show immunodiagnostic relevance of *Mtb* sensitization after exposure. Even though this phenomenon is subject to several potential clinical and immunological interpretations, some insights suggest the involvement of the innate immune response [8,19,20,21]. Generally, innate immunity fails to clear the infection in the majority of exposed humans, and *Mtb* dominates the initial stage of infection, taking advantage of phagocytes recruitment to infection sites for its expansion [22,23]. The bacteria can survive and replicate in infected cells via several immunosuppressive mechanisms, including inhibition of phagosome maturation, phagosome–lysosome fusion, production of reactive oxygen species, hamper major histocompatibility (MHC) class-II antigen presentation [21]. Additionally, *Mtb* manipulates death modalities of infected cells to improve cell-to-cell spreading [18,23]. These and other virulence factors contribute to the severe innate immune suppression in the lung and lead to the slowed-down recruitment and trafficking of dendritic cells (DC) to the drain lymph nodes. This peculiarity of *Mtb* infection results in a delayed onset of the adaptive immune response [24]. Moreover, DC migration from the primary site of infection to the bloodstream causes the colonization of previously uninfected lobes of the lung and the establishment of extra-pulmonary disease [18].

T-lymphocytes stimulated in the draining lymph nodes mediate the formation of the granulomas, primarily composed of macrophages, DCs, and T- and B-lymphocytes. *Mtb* containment within these lesions results in the development of latent TB infection (LTBI) [10]. In this stage, the individual becomes asymptomatic and cannot transmit the disease to healthy people. In the LTBI stage, a dynamic immunological equilibrium is established between the host and the pathogen. *Mtb* is prevalently in the dormant state, and its activities are markedly downregulated, while a subpopulation of bacteria is metabolically active and continues to replicate [18]. Hypothetically, the expression of a modified antigen repertoire causes the physiological shift of the bacteria into a non-replicative phenotype, as confirmed by human studies showing that the tubercular antigens recognized by healthy individuals with latent TB are different from those by active TB patients [25].

Approximately in 10% of the cases, reactivation of latent TB can progress to active, symptomatic disease [10]. This phenomenon is widely attributed to ‘weakened’ immunity, and that is the main reason for the emerging syndemic interaction between HIV and TB epidemics [26]. HIV-associated TB contributes substantially to the number of deaths, and it is estimated that people living with HIV are 15–22 times more likely to develop active TB than people without HIV [27].

## 4. Strategic Goals for New TB Vaccine Development

### 4.1. Target Population

One of the main challenges in developing a new TB vaccine is understanding who should be vaccinated and when. Therefore, host infection status required for efficacy (pre- or post-infection) and the desired effect type (prevention of infection or disease) represent essential factors to take into consideration in the design of a new vaccine [28]. Considering that the risk of *Mtb* infection and progression to TB disease increases with age, the WHO has proposed two sets of preferred product characteristics to guide vaccine candidate’s development, one addressing adolescents and adults, the other targeting neonates. Despite the development of a more effective vaccine than BCG would be desirable, it is expected that infant-targeted vaccination would require many years to become a cost-effective measure unless lifelong protection and high efficacy would be observed [9,29]. Children show lower rates of TB notifications, lower proportions of smear-positive pulmonary TB, and, consequently, they exhibited a smaller contribution to TB transmission [29]. Therefore, adolescents and adults should be defined as a priority strategic target as they represent the leading source of *Mtb* dissemination [9,30,31].

**Figure 2:**
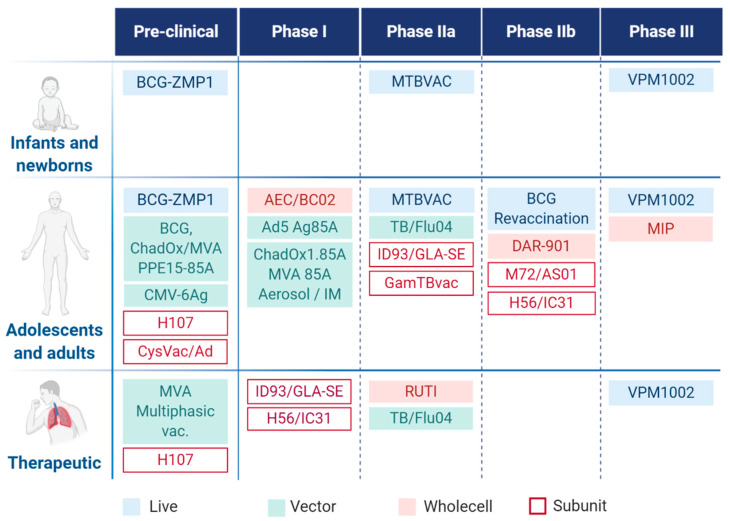
Tuberculosis vaccine pipeline with target population.

Novel TB vaccine candidates that enter clinical trials (Figure 2) can be categorized as follows: (i) preventive vaccines that are administered before *Mtb* infection—typically to neonates; (ii) booster vaccines, administered after previous BCG vaccination—typically to adolescents and adults with LTBI; (iii) post-exposure therapeutic vaccines drugs—to adults with to treat TB in adjunct with drug therapy [32].

### 4.2. Tuberculosis Vaccine Pipeline

Currently, 15 vaccine candidates are being investigated in active clinical trials (Figure 2), and additional candidates are tested in the pre-clinical phase. MVA85A (modified vaccine Ankara 85A) was the first booster vaccine candidate to enter vaccine efficacy evaluation since the introduction of BCG [33]. Unfortunately, the Phase IIb trial with previously BCG vaccinated healthy infants ended up with undesired results because the difference between the vaccinated and the placebo groups was not significant, and the interpretation was the absence of efficacy of MVA85. However, results were very informative to the scientific community, leading to a return to basic discovery with the primary goal to explore a greater diversity of approaches and develop better efficacy markers as well as improved in vitro and in vivo model systems [34,35].

In the recent vaccine pipeline, three live attenuated, four whole-cell or fragmented, three viral vectored, and five subunit-type vaccines are studied, of which six candidates enter the clinical IIb and III phases [36].

### 4.3. Subunit Vaccines against Tuberculosis

Traditionally, cell-based vaccines have been in the spotlight of vaccine development strategies because they generally produce long-lasting immunity. Nonetheless, they display several drawbacks, such as manufacturing difficulties, high feasibility costs, and critical stability. However, the main concern regards their safety as they may cause autoimmune or allergic reactions. Moreover, attenuation or inactivation of such vaccines might not be perfect, leading to the pathogen’s return to its virulent state, as it happened in the “Lübeck disaster” where 72 BCG-vaccinated children out of 252 died after developing pulmonary TB.

However, subunit vaccines, which consist primarily of proteins or peptides, can face limitations concerning immunogenicity. Therefore, they require immunostimulatory adjuvant or delivery systems to enhance the immunological response [37].

#### 4.3.1. Protein Antigens and Immunological Response

The development of an efficient vaccine requires not only accurate antigens choice but also activating the right ratio of a protective and suppressive immune response against the selected pathogen.

Even if intensive efforts have been underway, the identification of well-defined protective antigens has proved to be a challenging goal in TB subunit vaccine development.

While *Mtb* is a complex microorganism, composed of approximately 4000 genes, the selection of a small number of antigens among them is complicated because of the limited knowledge about the function of several antigens and their differential expression at different stages of the pathogen life cycle [31,38]. Moreover, *Mtb* escape mechanisms, as the downregulation of antigen processing and presentation, have made the identification procedure more difficult, resulting in the current lack of stringent immunological markers as correlates of protection [38,39].

The protein adjuvanted subunit vaccines that are currently under clinical evaluation use 12 mycobacterial antigens in different combinations and formulations (see Table 1).

Antigens involved in the active bacteria replication, such as Ag85, ESAT6, and CFP10, are extensively used in TB vaccination as they have proved to be highly immunogenic and showed protection in different animal models [41]. EsxV, EsxW, and TB10.4 are members of the Esx family and they are associated with the virulence of the bacteria as well. PPE18 and PPE42 belong to the so-called PPE family because of the presence of conserved Pro-Pro-Glu (PPE) motifs at the *N*-termini. As revealed by genome sequence analysis, genes encoding PPE proteins are immediately upstream of the Esx gene family, and they seem to play a role in driving localization or secretion of different proteins, such as ESAT6 [42,43]. On the other hand, Rv1813 and Rv2660 are stress-induced antigens associated with the dormant state of the pathogen. While the exact function of PepA, also called MTB32A antigen, is still unknown, although it seems to be a putative serine protease.

Several studies highlighted that these antigens are sharing a common characteristic, immunodominance. The immunogenicity of a protein antigen is heterogeneous; hence, some regions can elicit a more efficient expansion of T-cells than others can.

The current TB vaccine pipeline suffers the shortcoming of being focused on a narrow set of antigens characterized by suboptimal activity. Moreover, clinical trial data complicate to clarify the real effectiveness of these antigens’ subset and whether their intrinsic features represent an advantage for a TB vaccine candidate [7,38,41]. Therefore, the current strategy suggests to broad antigen repertoire, involving a more diversified array of antigens to improve the outcome.

The majority of vaccine candidates in the TB pipeline were selected based on their ability to induce interferon-gamma producing T-cells. However, while the protective role of CD4+ T-cells against *Mtb* is well-established; it seems evident that focusing on this classical T-cells subtype response is essential, but maybe not sufficient to obtain a fully protective response [8,38,39,44]. Notable attempts have focused upon identifying antigens capable of stimulating CD8+ T-cell mediated responses since *Mtb*-specific CD8+ T-cells have been detected in individuals with TB and LTBI as well [45].

Recently, the potential role of Donor Unrestricted T-cells (DURTs) is subject to increasing interest; it is a subset of T-cells that interact with human antigen-presenting cells (APCs) through non-MHC antigens presenting systems [8,46]. These non-conventional T-cells can recognize non-protein antigens, such as lipids and glycolipids that are part of the *Mtb* lipidome, which could be immunogenic to humans and may act as a booster of the immune response against the pathogen. Moreover, the multi-epitope approach is catching the eye of the scientific world as a promising strategy against different diseases, from oncology to infections, because it can expand the targeting spectra [47]. For example, it is possible to target several strains, different stages of the life cycle, or even distinct pathogens. The concept of targeting both the latent and the active state of the pathogen is not a novelty in TB vaccine design. Subunit vaccines ID93/GLA-SE and H56:IC31 are both candidates under clinical evaluation characterized by the presence of antigens associated with the virulence of the bacteria and the latency as well.

#### 4.3.2. Adjuvants and Formulation Strategies for Subunit Vaccines

**Figure 3:**
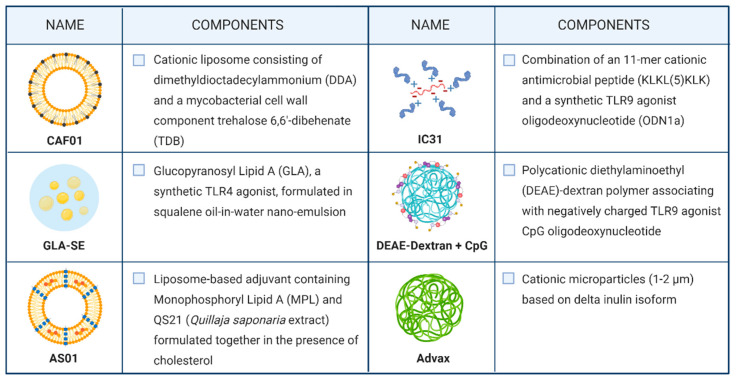
Vaccine adjuvant delivery system used in candidates undergoing clinical trials.

As previously mentioned, peptide- or protein-based vaccination is not free from challenges to overcome, such as low immunogenicity and bioavailability limitations. For this reason, a suitable formulation needs to be accurately designed. Nowadays, adjuvant and delivery systems are no longer mutually exclusive components in the field of vaccine development. Both of them can have the potential to adequately stimulate an immune response while simultaneously protecting the antigen degradation and transporting it to the desired tissue [48]. Indeed, most adjuvants effective in TB animal models, including the ones in clinical development, rely on vaccine adjuvant-delivery system (VADS) (Figure 3).

For decades alum was the only adjuvant used in licensed human vaccines, and it still represents a benchmark in this field. However, it has been considered unsuitable for vaccines against intracellular pathogens, like *Mtb*, because of its low ability to induce Th1-cellular immunity as well as CD8+ cytotoxic responses [49]. On the other hand, recent studies suggest that the modification of alum physicochemical properties, including the reduction of the particle size to the nanometer scale, can induce cellular immune responses characterized by Th1-cytokine secreting CD4+ T-cells [50,51].

Currently, the use of liposomes and emulsions as VADS is a prominent strategy used in TB vaccine formulation; examples include AS01, CAF01, and GLA-SE.

CAF01 is the first member of the Cationic Adjuvant Formulation (CAF) series developed by the Statens Serum Institut. This two-component liposomal adjuvant system is composed of a cationic liposome vehicle (*N,N*-dimethyl-*N,N*-dioctadecylammonium (DDA)) stabilized with a glycolipid immunomodulator (trehalose 6,6′-dibehenate (TDB)). While DDA alone can promote antibody-mediated and cellular immunological responses, it has shown intrinsic physical instability. Therefore, TDB incorporation act as a stabilizer of the system by enabling hydrogen bonding between the liposomal membrane and the surrounding water. Accordingly, CAF01 has been demonstrated as a highly stable formulation with an attested shelf life at 4 °C for more than two years [52]. From an immunological point of view, TDB is characterized by intrinsic immunostimulatory properties, as it is a synthetic analog of the cord factor (trehalose 6,6′-dibehenate (TDB)) located in the mycobacterial cell wall. Thus, it enhances the cellular immune response induced by DDA liposomes [53]. CAF01 has proved to promote a long-lived immunity with a Th1 profile in animal models and to elicit a Th17 response due to TDB signaling through the C-type lectin receptor Mincle. A recently conducted study on subcutaneous immunization with H56:CAF01 followed by an intranasal boosting did not skew the Th1/17 profile established by parenteral vaccine administration and did not confer additional control of pulmonary TB after aerosol *Mtb*-challenge [54]. However, a phase I clinical trial aimed to evaluate the CAF01 safety profile highlighted no concerns associated with the administration of the CAF01-adjuvanted vaccine to healthy adults (NCT00922363) [55].

Glucopyranosyl Lipid Adjuvant (GLA), a synthetic derivative of Monophosphoryl Lipid A (MPL) with TLR4 agonistic action, is formulated in a stable oil-in-water squalene emulsion (SE) to conceive GLA-SE. GLA is a fully synthetic derivative of lipopolysaccharide (LPS) that maintains mostly the immunostimulatory activity of the original molecule while it displays considerably reduced toxicity [56]. The combination of GLA-SE adjuvant and the fusion protein ID93 is currently under ongoing phase IIa clinical trial as a TB vaccine candidate, wherein its safety, immunogenicity, and efficacy are evaluated in previously BCG-vaccinated healthy healthcare workers. Phase I clinical trials previously conducted on healthy adult individuals highlighted the good tolerability of the adjuvant [57,58]. Furthermore, the inclusion of GLA-SE led to higher T-cell response magnitudes and to improve the overall T-cell response quality by eliciting a significantly higher proportion of polyfunctional CD4+ T-cells, producing TNF+, IL-2+, IFN-γ cytokines. Additionally, an increased antibody production, prevalently of IgG1 and IgG3 subclasses, was observed after ID93:GLA-SE vaccination [58].

M72:AS01E, one of the most advanced subunit vaccines in the pipeline was recently evaluated in a phase IIb clinical trial (NCT01755598), showing 54% efficacy in HIV-negative individuals with latent TB when administered intramuscularly in emulsion form [59]. Furthermore, the study evidenced that M72:AS01E vaccination protects against progression to pulmonary tuberculosis disease for at least three years [60]. AS01 is a liposome-based adjuvant that contains two immunostimulants, MPL and QS-21, formulated together in the presence of cholesterol to abrogate the haemolytic activity of QS-21. As mentioned above, MPL is a well-established pro-inflammatory adjuvant that directly activates APCs expressing TLR4 and stimulating NF-ĸB transcriptional activity. QS-21 is a natural saponin composed of a purified fraction of two isomeric triterpene glycosides from the tree *Quillaja saponaria*. While the exact mechanism of action of QS-21 has not been fully elucidated, the latest hypothesis suggested its action on both T-cells and APCs to induce a Th1 immune response [61]. The mechanism of action studies on QS-21 suggested the aldehyde group as the principal mediator of T-cell interaction and further activation, whereas QS-21 forms complexes with cholesterol to intercalate on the cell membrane of APCs. Moreover, it allows the early endosomal escape of the antigens for further processing inside the cell that results in peptides presented on the DCs surface to naïve CD8+ T-cells to yield cytotoxic T-lymphocytes [61].

A different approach was used in the development of the two-component adjuvant IC31, currently under clinical evaluation in combination with the fusion protein H56. IC31 is composed of the synthetic antimicrobial positively charged peptide KLK (KLKL_5_KLK) and ODN1a, a phosphodiester-backboned DNA oligonucleotide consisting of repeats of the dinucleotides deoxyinosine and deoxycytosine (poly I:C). KLK and ODN1a are formulated in a stoichiometrically fixed molar ratio of 25:1, respectively. While KLK is a poor adjuvant itself, its poly-cationic structure is suggested to play a crucial role in the delivery of ODN1a and the antigens to target APCs. It also induces depot formation after subcutaneous immunization, essential to provide constant antigen-specific stimulation of immune responses over a long period. ODN1a is an immunostimulatory molecule, acting through the TLR9/MyD88 pathway to promote Th1 biased immune responses [62]. H56:IC31 is currently under clinical evaluation both as a vaccine for adults and adolescents and as a therapeutic vaccine. However, the vaccine showed acceptable safety and tolerability profile in *Mtb*-infected and *Mtb*-uninfected adults as well as durable antigen-specific CD4+ T-cell responses [63,64].

Recently, polysaccharides are gaining attention as adjuvants because of their biocompatibility, biodegradability. Dextran is one of the most investigated α-glucan in drug and antigen delivery and has a long history of medical use because generally considered safe.

GamTBVac is a subunit vaccine under clinical development wherein a fusion protein with a dextran-binding domain is combined with a novel dextran/CpG adjuvant. The mixture results in nanoparticles consisting of DEAE-dextran core covered with a mixture of TLR9 agonist CpG through relatively week electrostatic interaction. The safety and immunogenicity of GamTBvac have been evaluated in a recently completed phase IIa trial in healthy BCG-vaccinated adults (NCT03878004), following successful clinical evaluation in a phase I trial in Russia [65].

Advax is a novel plant-derived adjuvant based on inulin delta isoform with the ability to form cationic particles of approximately 1–2 µm in diameter. Advax-based adjuvants have previously been proved to induce protective immunity against several pathogens in a wide range of animal species. Furthermore, this adjuvant is well-tolerated and immunogenic in human subjects. Currently, the use of Advax-based adjuvants in TB vaccine design is under pre-clinical evaluation. Recently, the Advax-based formulation of CysVac2 fusion protein has shown to confer protection against aerosol challenge at a comparable level to other TB vaccine candidates that have entered clinical trials, such as ID93/GLA-SE and M72/AS02. Moreover, the addition of CpG oligonucleotide, a TLR9 agonist, has resulted in enhanced immune cell recruitment and the subsequent multifunctional effector T-cell response [66,67].

#### 4.3.3. Peptide-Based Subunit Vaccines

Conversely, the peptide-based vaccine approach has become an attractive strategy in vaccine development because its manufacture is simple, fast, safe, reproducible, and cost-effective. Peptides are chemically well-defined compounds with good stability, and they do not rely on refrigerated storage. Overcoming the cold chain dependency is another advantage of the use of peptide-based vaccines. Besides, customizable design results in a reduced risk of harmful responses and side effects [48,68,69].

Numerous peptide-based vaccine candidates can be found in advanced clinical development targeting (i) infectious agents such as the highly polymorphic malaria, influenza virus, Hepatitis C virus, and HIV; (ii) Alzheimer’s disease where immunization with peptides could result in the clearance of neurotoxic forms of β-amyloid and Tau proteins and (iii) tumor cells [37]. The immuno-oncology approach to fighting against cancer and the use of peptides to provoke a sufficient T-cell response against tumor cells became one of the most promising measures of the decade [70,71,72].

While seven protein-based candidates are present in the TB vaccine pipeline [73], peptide-based vaccine candidates are being investigated only in pre-clinical studies yet (Table 2). For example, a synthetic long-peptide (SLP) derived from immunodominant proteins of *Mtb* showed protection in a murine model of tuberculosis [74]. Also, the immunization with self-adjuvating lipopeptide vaccine candidates is attracting more and more attention [75]. For example, Agrewala [76] focused on a self-adjuvating synthetic vaccine construct (L91). They recently proved that the conjugate is immunogenic in the murine and guinea pig models of TB and conferred better protection than BCG against *Mtb* [77]. They also showed that the combination of drug therapy and L91 (D-L91) significantly declined the bacterial load in *Mtb* infected animals [78]. Lately, self-assembled peptide nanofibers have received interest as vaccines and immunotherapies for several applications, including infectious diseases. A recently published study highlighted that peptide nanofiber vaccines in combination with BCG reduce bacterial load in the lungs of *Mtb*-infected mice and thus may be an effective strategy for boosting BCG-primed individuals [79]. Moreover, some studies have suggested that developing subunit vaccines that include classical T-cell epitopes derived from proteins that are expressed by the active and latent form of the causative agent may represent a new frontier in tuberculosis vaccine research [80,81]. In this context, several supportive tools are used to predict the most suitable epitopes to involve in the vaccine design, including immunoinformatic, genomic, and proteomic approaches [39,82,83,84].

## 5. Conclusions

Over the last decade, the TB vaccine pipeline has significantly progressed. Despite the advances made, several questions are still unsolved, such as the deeper understanding of *Mtb* pathogenesis and resistance strategies, as well as the immunological profile of BCG.

Despite BCG being developed over a century ago, there is still a lack in the full comprehension of its mechanism of action in preventing TB, as well as its off-target effect linked to protection against other infectious diseases. For example, increasing interest has been placed in its potential role in improving COVID-19 outcomes [90,91]. Though WHO officially disclaim the hypothesis of BCG protective effect against SARS-CoV-2, several surveys suggested a higher propensity of countries with no BCG vaccination coverage to be severely affected by the virus. On the other hand, these findings cannot provide definitive proof of causality because of some intrinsic bias, such as variances in the demographic and genetic structure of each country population, differences in non-pharmaceutical measures adopted (like quarantine and social distancing), and in diagnosis and reporting COVID-19 cases as well. Therefore, randomized controlled trials using BCG are under evaluation to endow the highest quality proof for the hypothesis that BCG may protect against COVID-19 [92,93,94,95]. Three clinical trials have been registered this year to evaluate the protective role of BCG vaccination against SARS-CoV-2 infection [96,97,98].

While primary TB has been extensively studied in humans and animals, post-primary TB is seldom recognized or studied. Nowadays, we have greater ability to manipulate vaccines to induce desired immune responses, including the adoption of the vaccine technology, the choice of the antigen and the adjuvant as well as the preferred route of administration.

The main concern is related to what type of immune response should be induced. Clinical trials are essential to answer this question as we are suffering from the lack of good biomarkers of protective immunity for adult pulmonary TB [99]. Moreover, we experienced disagreement in between animal TB models and between different human TB vaccine studies. This could reflect that a correlate of protection may be specific for each vaccine platform or may vary for different TB antigens. In this regard, standardization of vaccine study protocols and harmonization of animal and human studies could improve the discovery of the protection correlates [100].

On the other hand, the increasing number of subunit vaccines in the clinical phase has evidenced the proof of concept that protein-based antigens empowered by an adequate adjuvant can represent a promising immunization strategy against *Mtb*, but further studies are required to broaden the antigen repertoire, diversify the immune response, and find suitable adjuvant systems to develop a vaccine efficiently preventive TB infection, especially now that the outbreak of MDR-TB has made new struggles to control TB prevalence.

## Figures and Tables

**Table 1:** Mycobacterial antigens used in subunit-type vaccines being under clinical investigations.

Gene Name	Protein Name	TB Database ^1^	UniProt Entry ^2^	Protein Length (aa)
*pepA*	MTB32A	Rv0125	O07175	355
*PPE18*	MTB39A	Rv1196	L7N675	391
*PPE42*	PPE42	Rv2608	P9WHZ5	580
*fbpA*	Ag85A	Rv3804c	P9WQP3	338
*fbpB*	Ag85B	Rv1886c	P9WQP1	325
*esxA*	ESAT6	Rv3875	P9WNK7	95
*esxB*	CFP10	Rv3874	P9WNK5	100
*esxH*	TB10.4	Rv0288	P9WNK3	96
*esxV*	EsxV	Rv3619c	P0DOA7	94
*esxW*	EsxW	Rv3620c	P9WNI3	98
*Rv1813*	Rv1813	Rv1813	P9WLS1	143
*Rv2660c*	Rv2660c	Rv2660c	I6Y1F5	75

^1^ Protein identifiers found in the Mycobrowser database and website (https://mycobrowser.epfl.ch/) [40]. ^2^ Universal Protein Resource (UniProt) database (https://www.uniprot.org/).

**Table 2:** A selection of peptide-based subunit vaccines under pre-clinical investigation.

Protein * (Rv Code) Position	Peptide Sequence	Formulation	Immunization	Efficacy	Ref.
TB10.4 (Rv0288) 4–11 and Ag85B (Rv1886c) 280–294	IMYNYPAM and FQDAYNAAGGHNAVF	Self-assembling peptide nanofibres with KFE8 (FKFEFKFE) + Pam2Cys adjuvant	Intranasal boosting of BCG-primed C57BL6 mice	8-fold expansion in multifunctional CD8+ T cell populations in the lungs and 1.3 log10 CFU reduction in lung bacterial burden.	[79]
Ag85B (Rv1886c) 96–111 and Ag85B (Rv1886c) 241–256	QDAYNAAGGHNAVFN and PAFEWYYQSGLSIVMP	Covalently conjugated to RVG peptide	C57BL/6 mice, s.c. or intranasal immunization	Enhances antigen presentation and in vivo immunogenicity.	[85]
ESAT6 (Rv3875) 51–70	YQGVQQKWDATATELNNALQ	DDA/MPL-A/IL2 emulsion	B6CBAF1 mice, s.c. at the back 3x	Protective immunity against *Mtb* (around 1 log reduction in bacterial numbers).	[86]
ESAT6 (Rv3875) (1–15)	MTEQQWNFAGIEAAA	CAF01	CB6F1 mice, s.c. at the base of the tail	Vaccine-promoted polyfunctional T cell response.	[87]
acr (Rv2031c) 91–110	SEFAYGSFVRTVSLPVGADE	Covalently attached to Pam2Cys	BALB/c mice or Duncan-Hartley guinea pigs, i.p. or s.c. 2x	Significant protection against *Mtb* infection in guinea pigs and mice.	[76]
(Rv1733c) 57–84	IPFAAAAGTAVQDSRSHVYAHQAQTRHP	Synthetic long peptide (SLP) with CpG ODN1826 adjuvant	HLA-DR3 transgenic mice injected 3x s.c.	Induces protection against live *Mtb* challenge and has therapeutic effect when used as a boost a prior BCG vaccination.	[74]
Ag85B (Rv1886c) 239–247 and IniB (Rv0341) 33–45 and PPE68 (Rv3873) 127–136	KLVANNTRL and GLIDIAPHQISSV and FFGINTIPIA	Branched chain palmitoyl-peptide conjugate on Tuftsin (TKPKG) carrier	BALB/cmice injected 3-times s.c.	Significantly lower number of bacteria in the spleen after i.p. challenge with *Mtb*.	[80]
hsp65 (Rv0440) 3–13 and Ag85B (Rv1886c) 56–64 and 19 kDa (Rv3763) 51–61 and hsp16 (Rv2031c) 31–50 and (Rv1733c) 63–77	KTIAYDEEARR and PSMGRDIKV and KVVIDGKDQNV and LRPTFDTRLMRLEDEMKEGR and AGTAVQDSRSHVYAH	Recompinant polyepitope with CpG ODN1826 adjuvant	HLA-DR3 transgenic mice injected 3-times s.c.	Induces significant numbers (12.7%) of IFN- +/IL-2+/TNF+ CD4+ T-cells and reduce CFU in lungs of Mtb infected HLA-DR3 mice.	[88]
MPT64 (Rv1980c) 190–198 And (Rv0476) 1–6	FAVTNDGVI and f-MLVLLV	Peptide-pulsed BMDCs	C57BL/6 mice injected iv with 106 peptide-pulsed BMDC	Elicited an expansion of peptide-specific CD8+ T-cells in the spleen and the lung, and a significant protection against an intratracheal challenge with *Mtb*.	[89]

* Protein names are used in the format as they were found in the cited reference. RVG: a 29 amino acid peptide (YTIWMPENPRPGTPCDIFTNSRGKRASNG) derived from Rabies Virus Glycoprotein that binds to the α-7 subunit of nicotinic acetylcholine receptors (AchR) of neuronal cells. CpG ODN1826: a synthetic immunostimulatory oligonucleotide that contains unmethylated CpG dinucleotides (a known TLR9 agonist).
